# Disease activity and cognition in rheumatoid arthritis: an open label pilot study

**DOI:** 10.1186/ar4108

**Published:** 2012-12-04

**Authors:** Graham Raftery, Jiabao He, Ruth Pearce, Daniel Birchall, Julia L Newton, Andrew M Blamire, John D Isaacs

**Affiliations:** 1 Department of Rheumatology, City Hospitals Sunderland NHS Trust, Kayll Road, Sunderland, SR4 7TP, UK; 2 Institute of Cellular Medicine, Newcastle University, Framlington Place, Newcastle-upon-Tyne, NE2 4HH, UK; 3 Institute of Ageing and Health and NIHR Biomedical Research Centre in Ageing, Campus for Ageing and Vitality, Newcastle-upon-Tyne, NE4 5PL, UK; 4 Neuroradiology Department, Regional Neurosciences Centre, Queen Victoria Road, Newcastle-upon-Tyne, NE1 4LP, UK; 5 Musculoskeletal Unit, Newcastle-upon-Tyne Hospitals NHS Foundation Trust, Freeman Road, Newcastle-upon-Tyne, NE7 7DN, UK

## Abstract

**Introduction:**

We hypothesised that fatigue in rheumatoid arthritis (RA) is related to TNF-alpha induced dysregulation of cerebral blood flow. Our objectives were to assess fatigue, cognitive function and cerebral blood flow before and after initiation of anti-TNF treatment.

**Methods:**

In a pilot study, 15 patients initiating treatment with adalimumab were assessed for fatigue using a visual analogue scale (FACIT-F), cognitive function using a panel of psychometric tests and regional cerebral blood flow using MR perfusion imaging.

**Results:**

Patients improved clinically after anti-TNF therapy in terms of DAS28 and FACIT-F. Furthermore significant improvements were documented in full scale, verbal and performance IQ following therapy. There was a non-significant trend towards reduced cerebral perfusion in both grey and white matter, and fatigue at 3 months correlated with cerebral blood flow in white (p = 0.014) and grey (p = 0.005) matter.

**Conclusions:**

We demonstrate for the first time a significant improvement in cognitive function following effective treatment of RA. Although we observed minor reductions in cerebral blood flow, and a correlation between cerebral blood flow and fatigue, a larger, controlled study would be required to affirm a causal relationship.

## Introduction

Patients with rheumatoid arthritis (RA) report reduced health-related quality of life, which is attributable to fatigue, pain and impairment of physical function. The fatigue experienced is a pervasive symptom, which patients consider highly important [1]. It is a different experience to normal tiredness; patients frequently describe overwhelming exhaustion as well as cognitive fatigue, hindering clear thought and concentration [2]. Effective treatment of RA, particularly with biologic drugs, improves fatigue but it is not clear if it also improves cognition [3]. While the improvement in fatigue is assumed to be a direct consequence of cytokine reduction, the physiological substrate for such a profound effect is unclear.

TNF has been implicated in a number of neuropathologies [4]. A previous study into the effects of TNF on the brains of Wistar rats found that a single intrastriatal bolus of TNF led to significant reductions (15 to 30%) in cerebral blood volume, which was dependent on TNF type-2 receptor activation, and was preventable with an endothelin receptor antagonist [5]. Neuroimaging studies in RA have identified hypoperfusion of the frontal and parietal lobes, while in systemic lupus erythematosus, hypoperfusion of the frontal lobes has been associated with cognitive dysfunction [6]. If TNF influences cerebral blood flow (CBF) in humans, then the chronically high levels associated with active RA may be implicated in cognitive impairment. We therefore hypothesised that treatment of active RA, particularly with TNF blockade, would lead to improvements in both fatigue and cognitive function, and that these effects would be related to changes in CBF. A small pilot study was initiated to address this possibility. Advances in magnetic resonance imaging (MRI) technology and scanning techniques have allowed direct and non-invasive imaging of CBF without the need for contrast injection. We applied a CBF MRI technique in a cohort of RA patients about to commence a TNF antagonist, and measured CBF, disease activity, fatigue and cognitive function before and during treatment.

## Materials and methods

This was an open-label pilot study. Cerebral MRI scans, fatigue scores, 28-joint disease activity score (DAS28), and psychometric assessment were performed on patients before, and 12 weeks into, anti-TNF therapy for RA. Ethical approval was granted by Newcastle and North Tyneside 2 Research Ethics Committee. Funding was provided by Abbott Laboratories. The funder was not involved with study design, performance or data analysis.

### Patients

Fifteen patients with RA according to 1987 American College of Rheumatology (ACR) criteria were recruited. Each had been identified as requiring anti-TNF therapy as part of routine clinical care. Patients were excluded if they had previously received any anti-TNF therapies or if they had contra-indications to undergoing cerebral MRI. Adalimumab 40 mg was administered by subcutaneous injection every 2 weeks. The patients otherwise received routine clinical care. The study was approved by the local research ethics committee and all patients gave written, informed consent.

### MRI

Co-registered conventional MR images were acquired on a Philips 3 Tesla scanner to define anatomy and show any areas of focal abnormality (3-dimensional T1-weighted sequence, TR/TE = 9.6/4.6 ms, 1 mm isotropic resolution; T2-weighted sequence, TR/TE = 7079/100 ms, 1 mm resolution, 3-mm slices). Slice orientation and the total volume of tissue sampled were standardised across all subjects. CBF imaging used a multi-slice pulsed arterial spin labelling (ASL) sequence, flow-sensitive alternating inversion recovery (FAIR) [7]. In-plane resolution was 4 mm with 12 contiguous 6-mm-thick slices (TR was set to 4 s, inflow time was set at 1.7 s and 40 image-pairs were collected, 25 minutes total imaging time for perfusion and associated T1 mapping data for absolute quantitation). Slice angle and extent were controlled to match the slices to the anatomical data.

### Psychometric testing

All patients completed a set of neuropsychometric tests at baseline and again after 12 weeks of treatment with adalimumab. The tests used were chosen to minimize any learning effect and each session was administered by the same trained researcher to avoid discrepancies in scoring. The selected tests (see below) were considered those most appropriate for patients with fatigue.

#### Assessment of global cognitive function: full-scale intelligence quotient

The full-scale intelligence quotient (IQ) provides a summary of overall cognitive ability and was obtained using a shortened version of the Wechsler Adult Intelligence Scale III (WAIS-III), which assesses vocabulary comprehension, visuoconstructive ability (block design), verbal reasoning (similarities) and nonverbal deductive reasoning (matrices). Raw scores are compared with population age and sex normative values, resulting in a T-score that is standardised to a mean of 100 and SD of 15.

#### Assessment of specific cognitive deficits using the WAIS-III

Three subtests of the WAIS-III were used to obtain non-standardised raw scores. The digit span test assesses immediate recall for sequences of numbers increasing in magnitude. The digit symbol search requires participants to indicate whether one of two symbols is present in an array; this depends on paired associate learning (the ability to deal with two stimuli at once and to be able to associate them). The patients also completed a trail-making test of visual scanning, requiring a combination of information-processing speed, manual motor speed, and sustained attention.

### Fatigue

Fatigue was measured using the functional assessment of chronic illness therapy fatigue scale (FACIT), a simple-to-administer questionnaire, which has been validated in RA patients [8]. The range of possible scores is 0 to 52; greater scores reflect less fatigue.

### MRI analysis

Perfusion images were processed into maps of quantitative regional cerebral blood flow (rCBF) and corrected for image distortion. The rCBF maps were analysed in two ways: (1) analysis of rCBF in grey and white matter was carried out to look for global differences between patients in brain perfusion, and for changes with therapy. The anatomical images were subjected to supervised tissue classification to define gross regions of interest (ROI) for white matter, grey matter, cerebro-spinal fluid (CSF), deep grey structures and any focal abnormalities. These ROIs were applied to the CBF dataset to determine average flow in each tissue type; (2) statistical analysis by group was applied to look for any regional differences or changes in perfusion. The Statistical Parametric Mapping (SPM) software package (SPM 5) was used to transform CBF data into a standardised brain space [9]. SPM was then applied to calculate pixel-wise group statistics between patients pre- and post-therapy to determine areas of differences in rCBF, and to perform regression analysis of clinical measures [10].

### Statistical analysis

Statistical analysis was performed using SPSS (Version 15 - IBM Corporation, USA). Tests for normality were performed using the Kolmogorov-Smirnov test. Interval data were compared using Pearson's correlation coefficient, and ordinal data were analysed using Spearman's correlation coefficient. Cognitive function and CBF at baseline and 12 weeks were compared using the paired *t*-test. DAS28 scores and FACIT fatigue were compared using the Wilcoxon signed-rank test. Possible predictors of cognitive function were analysed as independent variables using a forced entry method of multiple regression analysis. A *P*-value < 0.05 was considered statistically significant.

## Results

Fifteen patients were recruited and thirteen completed all aspects of the study (Table 1). One patient inadvertently commenced adalimumab prior to baseline MRI, and one patient stopped anti-TNF therapy after 6 weeks and declined further study participation. Analysis was based on the paired datasets from the 13 patients completing the study; 12 patients were female and one was male; the mean age of the group was 48.92 (SD 8.04) years.

**Table 1:** Patient demographic data, disease activity scores (DAS28), and fatigue scores (FACIT fatigue)

Patient number	Age (years)	Rheumatoid factor status	Disease duration (years)	Concurrent DMARD	Concurrent corticosteroid NSAID or COX2	DAS 28 pre-adalimumab	DAS 28 after 3 months of adalimumab	FACIT fatigue pre-adalimumab	FACIT fatigue after 3 months of adalimumab
1	49	+	13	leflunomide	diclofenac	7.38	2.84	15	33
2	43	+	7	none	celecoxib	7.42	3.88	2	46
3	57	-	14	Methotrexate leflunomide	celecoxib	6.37	3.41	22	45
4	60	+	4	none	celecoxib	6.32	2.59	46	44
5	44	-	9	none	none	6.36	4.36	9	25
6	37	+	17	methotrexate	naproxen	6.33	4.50	11	20
7	39	+	13	methotrexate	diclofenac	6.56	3.53	17	43
8	39	-	18	methotrexate	indomethacin	6.25	4.46	25	33
9	61	-	12	none	none	6.01	2.16	26	47
10	52	-	12	sulfasalazine	none	7.37	2.95	18	39
11	49	-	13	methotrexate	none	6.29	5.41	16	36
12	53	+	10	leflunomide	diclofenac	8.20	4.49	15	24
13	53	+	13	none	none	5.11	5.11	40	41

COX2, Cyclooxygenase-2 inhibitor; DMARD, disease-modifying anti-rheumatic drug; NSAID, non-steroidal anti-inflammatory drug.

### Clinical response

All participants had a high level of baseline disease activity and fatigue (table 1). There were significant improvements in DAS28 (*P *= 0.002) and FACIT F score (*P *= 0.002) with adalimumab treatment. A significant relationship was observed between age and baseline FACIT fatigue score (*P *= 0.03), increasing age corresponding with lower levels of fatigue, as well as between baseline DAS28 and baseline FACIT fatigue score (*P *= 0.018) (higher DAS28 corresponding with greater fatigue).

### Cerebral imaging

There was no significant influence of adalimumab treatment on either grey or white matter CBF (*P *= 0.183 and *P *= 0.210 respectively, data not shown). SPM software applied to calculate group statistics between patients pre- and post-therapy did not identify any focal areas of statistically significant differences in rCBF.

**Figure 1:**
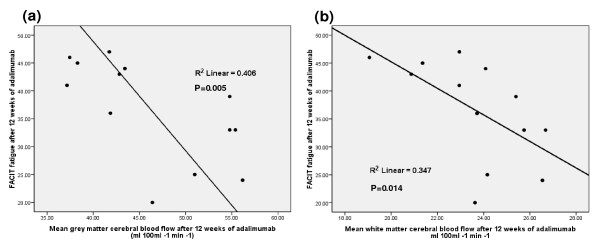
**Correlation between fatigue levels and cerebral flow after 12 weeks of treatment with adalimumab**. (**a**) Grey matter (*P *= 0.005). (**b**) White matter (*P *= 0.014). FACIT, functional assessment of chronic illness therapy fatigue scale.

FACIT fatigue score after 3 months of treatment correlated with both grey (Correlation coefficient (ρ) = -0.724, *P *= 0.005) and white (ρ = -0.660, *P *= 0.014) matter CBF at 3 months, lower CBF corresponding to less fatigue at 3 months (Figure 1).

### Cognitive function

**Figure 2:**
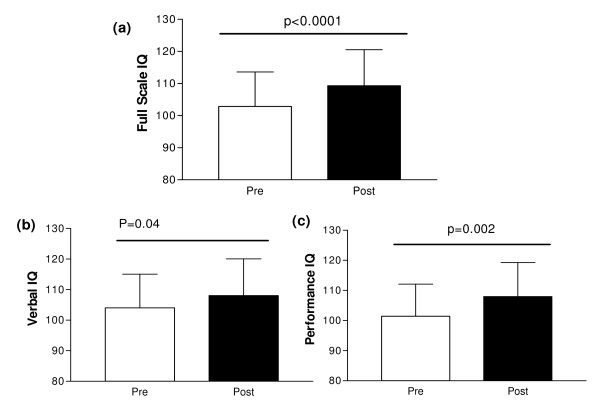
**Cognitive function test changes between baseline and 12 weeks**. (**a**) Full scale IQ. (**b**) Verbal IQ. (**c**) Performance IQ. Standardised data presented. Pre, pre-treatment with adalimumab; post, post-treatment with adalimumab.

The mean full scale IQ prior to anti-TNF therapy was 103.8 (SD 10.6), which compares to an age- and sex-adjusted population mean score of 100 (SD 15). Significant improvements in cognitive function were associated with anti-TNF therapy (Figure 2); raw and normalised scores for full scale IQ, performance IQ, and verbal IQ improved significantly after 12 weeks of treatment. Particularly marked improvements were seen in performance IQ (raw data *P *= 0.004, normalised data *P *= 0.002). Further analysis to investigate potential correlates of IQ improvements did not reveal any significant associations with FACIT fatigue, DAS28, or CBF.

## Discussion

To our knowledge this is the first study to demonstrate an improvement in cognitive function following effective treatment of RA. Improvements in cognitive function related particularly to performance IQ, but we did not demonstrate a relationship with CBF, fatigue or disease activity. The physiological correlate of improved cerebral function therefore remains unexplained and requires further study. Although improvements followed treatment with anti-TNF therapy, the lack of a control group in this small pilot study preclude any conclusions being drawn regarding the specificity of the effect, and future studies should incorporate additional treatment options.

There is a paucity of data on cognitive function in RA populations. Appenzeller, *et al. *observed cognitive impairment in 30.0% of RA patients and 7.5% of healthy controls [11]. Patients with RA had worse outcomes in verbal fluency, logical memory and short-term memory, while those with active disease had worse cognition scores. A psychometric assessment of 30 patients with RA, which related performance to neuroimaging, found an abnormal prevalence of impaired cognitive performance, particularly in attention tasks, planning abilities and cognitive flexibility, and identified deficits related to cortical function; visual memory was impaired in 50% and verbal memory in 35% [12]. Abnormalities on MRI were found with white matter hyperintensity in 35% of patients, all of whom had low scores in attention, executive and visuospatial tests.

Fatigue is virtually ubiquitous in RA, part of which is a cognitive fatigue with lack of clarity of thought and an inability to concentrate [2]. It often improves with biologic therapies, suggesting that aspects of fatigue are centrally driven and mediated by TNF or interleukin-6 [3,13]. While pain and depression may underpin some of this relationship, fatigue is present in as many as one third of RA patients with low levels of disease activity and lack of clinical depression [14]. Our work reaffirms that the relationship between fatigue, cognition and disease activity is a complex one, and that some aspects are responsive to therapy. The improvement in performance IQ suggests that cognitive function alone needs to be carefully considered; it may be independent of fatigue or it may be a subset of the symptom complex of fatigue, with the exciting prospect that aspects of it are modifiable by treatment.

MRI did not reveal a physiological correlate of changes in cognitive function, although fatigue at 3 months correlated with CBF in white and grey matter. We applied a pulsed ASL sequence technique (FAIR), which allowed a noninvasive measurement of perfusion. This is the first use of ASL in an RA cohort. Bartolini, *et al. *assessed perfusion with single-photon emission computed tomography and found 85% of patients had hypoperfusion of the frontal lobes, with parietal lobe hypoperfusion in 40%. Our study was designed specifically to detect longitudinal changes in CBF as a function of treatment, and so does not allow cross-sectional quantitative assessment of baseline regional perfusion differences. However, visual inspection of the CBF data did not reveal any obvious frontal hypoperfusion in our group. Interestingly, lower CBF correlated with lower levels of fatigue at 3 months, alongside a weak trend for a reduction in CBF after 3 months of treatment with adalimumab. These data are counterintuitive; our hypothesis was that TNF blockade would enhance CBF and this enhancement would correlate with improvement in fatigue. However, we may have missed any acute effects of treatment on CBF and would recommend imaging soon after treatment initiation in future studies. Nonetheless, the inverse correlation between fatigue and CBF at 3 months remains intriguing.

## Conclusions

Our small pilot study has demonstrated an apparent improvement in cognitive function with effective treatment of RA. We could not link changes in CBF with improved cognitive function despite a possible influence on fatigue. Future studies need to address and confirm the specificity of this treatment effect, as well as seeking earlier treatment-related changes in CBF. The major challenge is to build a template for cognition and fatigue in RA, with a view to establishing a more complete understanding of pathophysiological influences, with the ultimate aim of identifying and directing effective treatment.

## Abbreviations

ACR: American College of Rheumatology; ASL: arterial spin labeling; CBF: cerebral blood flow; CSF: cerebrospinal fluid; DAS28: 28-joint disease activity score; DMARD: disease-modifying anti-rheumatic drug; FACIT-F: functional assessment of chronic illness therapy fatigue scale; FAIR: flow-sensitive alternating inversion recovery; IQ: intelligence quotient; MRI: magnetic resonance imaging; NSAID: non-steroidal anti-inflammatory drug; RA: rheumatoid arthritis; rCBF: regional cerebral blood flow; ROI: region of interest; SPM: Statistical Parametric Mapping; TNF: tumour necrosis factor; WAIS-III: Wechsler adult intelligence scale III.

## Competing interests

JDI received a speaker fee from Abbott in 2010. Other authors have no financial competing interests.

## Authors' contributions

GR, AMB, JLN and JDI were involved in study design. GR, JH, RP, JLN, AMB and JDI collected and analysed data. GR, AMB, JLN and JDI wrote and edited the manuscript. DB reviewed MRI scans. All authors have read and approved the manuscript for publication.
